# Personalizing Oncolytic Virotherapy for Glioblastoma: In Search of Biomarkers for Response

**DOI:** 10.3390/cancers13040614

**Published:** 2021-02-04

**Authors:** Eftychia Stavrakaki, Clemens M. F. Dirven, Martine L. M. Lamfers

**Affiliations:** Department of Neurosurgery, Brain Tumor Center, Erasmus University Medical Center, 3015 CN Rotterdam, The Netherlands; e.stavrakaki@erasmusmc.nl (E.S.); c.dirven@erasmusmc.nl (C.M.F.D.)

**Keywords:** oncolytic viruses, glioblastoma, clinical trials, biomarkers, personalized oncolyticvirotherapy

## Abstract

**Simple Summary:**

Glioblastoma (GBM) is the most frequent and aggressive primary brain tumor. Despite multimodal treatment, the prognosis of GBM patients remains very poor. Oncolytic virotherapy is being evaluated as novel treatment for this patient group and clinical trials testing oncolytic viruses have shown impressive responses, albeit in a small subset of GBM patients. Obtaining insight into specific tumor- or patient-related characteristics of the responding patients, may in the future improve response rates. In this review we discuss factors related to oncolytic activity of the most widely applied oncolytic virus strains as well as potential biomarkers and future assays that may allow us to predict response to these agents. Such biomarkers and tools may in the future enable personalizing oncolytic virotherapy for GBM patients.

**Abstract:**

Oncolytic virus (OV) treatment may offer a new treatment option for the aggressive brain tumor glioblastoma. Clinical trials testing oncolytic viruses in this patient group have shown promising results, with patients achieving impressive long-term clinical responses. However, the number of responders to each OV remains low. This is thought to arise from the large heterogeneity of these tumors, both in terms of molecular make-up and their immune-suppressive microenvironment, leading to variability in responses. An approach that may improve response rates is the personalized utilization of oncolytic viruses against Glioblastoma (GBM), based on specific tumor- or patient-related characteristics. In this review, we discuss potential biomarkers for response to different OVs as well as emerging ex vivo assays that in the future may enable selection of optimal OV for a specific patient and design of stratified clinical OV trials for GBM.

## 1. Introduction

Oncolytic viral therapy or virotherapy is a form of immunotherapy showing promising results for cancers with poor prognosis [1]. In this approach, oncolytic viruses (OVs) are employed to kill tumor cells, while in parallel stimulating an anti-tumor immune response [2]. OVs exhibit either natural tropism to malignant cells or their genome is altered to confer them higher specificity for malignant cells [3]. Viruses from ten different families (Adenoviridae, Herpesviridae, Paramyxoviridae, Reoviridae, Retroviridae, Picornaviridae, Parvoviridae, Poxviridae, Rhabdoviridae, Alphaviruses) have thus far been utilized as oncolytic virus platforms in clinical trials for various cancer types [2].

One deadly type of cancer is glioblastoma multiforme (GBM), the most common and aggressive primary brain tumor [4]. The standard treatment consists of maximal safe surgical resection followed by radiotherapy plus concomitant and adjuvant temozolomide chemotherapy. However, the median overall survival among all GBM patients is less than one year, and only 15 months in patients receiving complete standard treatment with 3-year survival being less than 10% [5,6]. In the past decades, numerous therapeutic approaches have been tested in clinical trials, with disappointing outcomes. The main obstacles in treating GBM include its infiltrative growth, its intrinsic resistance to chemo- and radiotherapy, its notorious intratumoral heterogeneity with dynamic changes in subclones facilitating treatment escape, its protected location behind the blood-brain-barrier and the immunological ‘cold’ microenvironment of these tumors. These hurdles to more conventional therapies, as well as the dismal prognosis of GBM patients, have encouraged scientists and clinicians to develop and evaluate the local application of various types of oncolytic viruses in this patient group. Table 1 summarizes the most commonly applies OVs in GBM trials. The OVs differ in their primary attachment molecules to host receptors as well as in the source of their tumor selectivity, which may be derived from a natural tropism to cancer cells or by genetic engineering.

In a recent review, Chiocca et al. summarized the findings [19] from all the recent GBM oncolytic virotherapy trials and illustrated that a subgroup of GBM patients responds exceptionally well to OV treatments, with survivors at 36-months, and with some patients exhibiting long term remission [20,21]. This phenomenon has also been observed in OV trials for other cancer types. For instance, a phase II clinical trial employing an oncolytic herpes simplex virus 1 for stage IIIC or IV melanoma showed 26% overall response [22].

These observations raise the question: would the responding patients have been the same individuals if they had been treated with any other OV, or are we looking at responders to a specific OV? In other words, is the elicited immune response a generalized one for all types of OVs, or does each OV elicit a specific anti-tumor immune response? The latter would suggest that response rates may be significantly increased if we are able to define which OV is best suited for a particular patient. Identification of robust predictive biomarkers for OV response would allow future design of stratified clinical trials employing multiple OV strains. The replication efficiency of the virus is thought to be of importance for generation of the subsequent inflammatory and anti-tumor responses. Moreover, host immune status is also expected to contribute to the efficacy of OV treatment. This review, therefore, focuses on tumor and host resistance mechanisms to viral infection, replication and oncolysis and discusses potential biomarkers that have previously been reported in relation to sensitivity or resistance to the most frequently employed OVs in preclinical and clinical GBM research.

## 2. Glioblastoma

### 2.1. Heterogeneity, Stem Cells and Therapy Resistance

Common molecular abnormalities involved in the evolution of glioblastomas include aberrations in the oncogenes (EGFR, PDGF and its receptors) and tumor suppressor genes (p16INK4a, p14ARF, PTEN, RB1, and TP53), which are often observed in other human cancers as well [23]. GBM is also characterized by inter-tumoral heterogeneity, which is highlighted by the classification of GBMs into three subgroups: proneural, classical and mesenchymal [24,25]. Each subtype is characterized by specific gene expression patterns and molecular abnormalities, resulting in different clinical treatment outcomes [25,26]. Proneural subtype has the most favorable prognosis among the three subtypes; aberrations in the isocitrate dehydrogenase 1 (IDH1) gene and the platelet-derived growth factor receptor A (PDGFRA) define this subgroup. The classical subgroup is characterized by the amplification of EGFR, lack of TP53 mutations and often with homozygous CDKN2A deletions [26]. Lastly, the mesenchymal subtype is the most aggressive and it is characterized by aberrations in the neurofibromin 1 (NF1) and PTEN genes [23]. It is also characterized by a pro-inflammatory environment compared with the other subtypes [27]. It was hypothesized that one underlying cause for this was the higher incidence of tumor-associated antigens (TAAs), however this could not be proven, as specific tumor antigens are expressed in each subtype [27]. Nevertheless, this classification has not led to altered or adapted treatment approaches [28,29].

Apart from intertumoral heterogeneity, intra-tumoral heterogeneity poses another therapeutic obstacle in treatment of GBM, allowing escape of subclones from (targeted) therapies and driving treatment resistance. This heterogeneity was captured by genome-wide and single cell RNA studies, which showed tumor cells with different transcriptional profiles within the same tumor [30,31]. In addition, it was shown that within the same tumor, different subtypes can coexist, highlighting the heterogeneity that characterizes GBM [31]. In another study, paired primary and recurrent tumor tissue samples were analyzed to determine the persistence of possible drug targets. The results showed that the molecular targets between primary and recurrent tumors changed by 90% [32]. This may explain the failure of drugs that target specific molecular mutations in GBM, such as the EGFR [33].

Eventually, most of the patients experience tumor relapse due to therapeutic resistance [29]. This therapeutic resistance is mainly attributed to glioblastoma stem cells (GSCs), which activate DNA repair mechanisms to promote survival after chemo- and radiotherapy [34]. Additionally, outgrowth of resistant subclones and downregulation of targeted molecules contribute to drug resistance. Furthermore, the highly infiltrative nature of GSCs makes total surgical resection of the tumor impossible [35]. The remaining and/or treatment-resistant clones will eventually generate functional vessels for the nutrient transport and develop tumor recurrence [34].

### 2.2. GBM Microenvironment: Local Immunosuppressive Mechanisms

Glioblastoma arises in the central nervous system (CNS) [36], which is an immunologically distinct site. In the past, the CNS was considered an immune privileged site, due to its unique properties. For instance, the blood brain barrier, which tightly regulates the transportation of the immune cells from the periphery to the CNS; the lack of antigen presenting cells in a non-inflamed state; and more importantly the lack of a classic lymphatic system [37,38,39]. The concept of CNS being immune privileged has now been revised. Recent studies have shown that antigens derived from the CNS can efficiently elicit an immune response [40]. More importantly, Louveau et al. [41] discovered a functional lymphatic system, parallel to the dural sinuses, a possible route of transportation of antigen-presenting cells to the deep cervical lymph nodes, where they can present CNS-derived antigens and prime T cells. These recent studies have provided evidence that CNS-derived antigens can mount a vigorous immune response, offering ground to investigate immunotherapy approaches for GBM.

The GBM environment is characterized by the high influx of tumor-associated macrophages (TAMs). In a non-inflamed state, the myeloid composition of the CNS consists of the tissue-resident macrophages that arise from the yolk sac, the microglia [42]. However, in GBM, the microenvironment is comprised mainly of a mixture of microglia and infiltrating monocytes from the periphery. Glioma cells produce a milieu of monocyte chemoattractant proteins along with other factors, leading to disruption of the blood-brain barrier and facilitating recruitment of monocytes from the periphery [43]. When monocytes arrive at the tumor site, glioma cells drive their polarization to an immunosuppressive M2 phenotype [44,45]. These M2-like TAMs promote tumor growth and migration as well as the immune invasion by hampering the adaptive immunity [44,46,47]. TAMs are the most abundant immune cell population in GBM and can consist up to 50% of the GBM tumor mass. Their importance in tumor growth is highlighted by the correlation between increased TAM numbers and worse prognosis in GBM patients; furthermore, TAM infiltration has been associated with the mesenchymal subtype of GBM, being the most aggressive one [48,49].

Another feature that facilitates the local immune suppression in GBM is T cell dysfunction. Severe T cell exhaustion is observed in GBM, which is characterized by upregulation of expression of co-inhibitory molecules like PD-1, LAG-3 and TIM-3 [50]. Furthermore, an increase in numbers of the regulatory T cells (Tregs), which can suppress the antigen-specific T cells, was found in high grade gliomas compared to low grade gliomas [51]. The recruitment of Tregs at the tumor site is mainly facilitated by the production of the attractant indoleamine 2,3 dioxygenase (IDO) by gliomas [52]. Another facet that contributes to the ‘’cold’’ tumor microenvironment is the relatively low mutational burden of GBM cells, associated with limited expression of neoantigens [53,54]. Taken together, GBM has all the characteristics of a tumor with low immunogenicity. The M2-like macrophages that are abundant at the tumor site, the dysfunctional T cells and the low neoantigen expression are some of the barriers that we need to overcome to design successful immunotherapies.

Considering all of the above, a therapeutic strategy that is not hindered by specificity for a single molecular target or differentiation state of tumor cells, that is delivered locally in a single surgical intervention, hence bypassing the BBB, that is self-perpetuating in its anti-tumor activity, and which can overcome the immune-suppressive tumor microenvironment, may offer opportunities for achieving therapeutic responses in glioblastoma patients. Oncolytic viruses offer such a treatment strategy.

## 3. Factors Affecting OV Therapy in GBM

**Figure 1:**
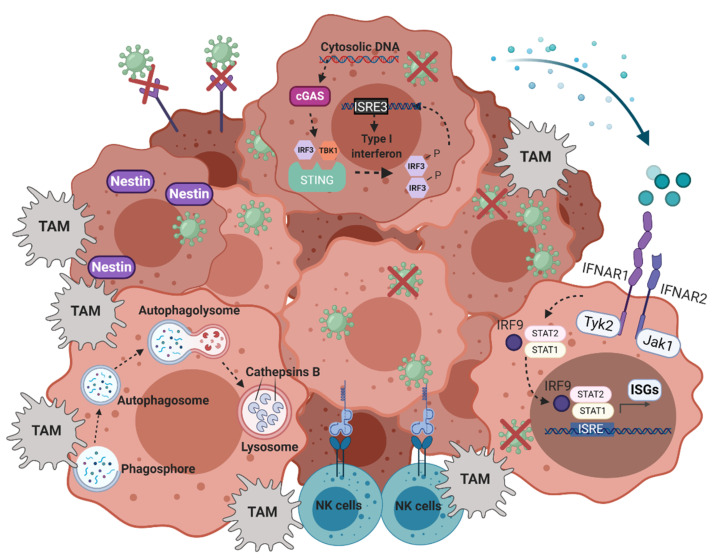
OV restriction mechanisms of GBM tumors. Infiltration of NK cells and tumor-associated macrophages (TAMs) at the tumor site, activation status of autophagy, expression of viral entry molecules and viral sensors (e.g., cGAS-STING) that lead to constitutive active type I interferon pathways, all could hamper the OV replication and oncolysis. Furthermore, cathepsin B expression and expression of specific proteins that drive specific tumor replication (e.g., nestin) could determine the OV efficacy. Created with BioRender.com.

Several oncolytic virotherapy clinical trials have shown impressive and durable responses in a subset of patients, indicating that OVs might be a very promising therapeutic tool for treating GBM. The establishment of an efficient viral infection, lysis of tumor cells, viral spreading and anti-tumor immune activation, all depends on multiple factors (Figure 1). It is therefore conceivable that we may improve OV efficacy if we take these factors into account when selecting patients for treatment.

### 3.1. Viral Entry Molecule Expression

Tumor cell infection and oncolysis are a prerequisite for mounting an inflammatory response in the tumor microenvironment and ultimately generating an anti-tumor immune response. This depends on the cell entry possibilities for the virus. As GBM cells are not the natural host cells for entry of most viruses, low levels or even lack of receptor molecules on these cells can form the first obstacle to virotherapy. It has been shown that tremendous inter-tumoral variability exists in expression levels of specific adenovirus and reovirus entry molecules on patient-derived GBM cells [55,56]. As a result, various retargeting strategies have been applied to overcome such limitations, including EGFR and integrin retargeting of OVs [57]. Therefore, OV efficacy could potentially be enhanced by stratification of patients based on expression of specific viral receptor molecules in their tumors.

### 3.2. Status of Oncogenic Signaling Pathways Affected in Glioblastoma

Many OVs applied in glioma studies are genetically engineered or have naturally evolved to exploit oncogenic signaling pathways in cancer cells, such as the Ras, Rb, p53 or nucleotide synthesis pathways [58]. Therefore, OV efficacy could potentially be enhanced by stratification of patients based on activation status or presence of mutations in targeted pathways.

Another targeting approach is by the insertion of tumor-specific promoters to drive specific viral replication in tumor cells and avoid toxicity to normal tissue [59]. Various promoter candidates have been applied to design tumor-specific promoter-driven OVs, including nestin, survivin, cyclooxygenase-2 (COX-2), C-X-C chemokine receptor type 4 (CXCR4), hypoxia inducible factor-1 (HIF-1) and telomerase [10,60,61,62,63]. Considering the intertumoral heterogeneity in transcription profiles of GBM, it would be expected that the response to such OVs might vary between GBM subtypes. One could hypothesize that GBM with proneural features might be more sensitive to viruses targeting cells expressing neuronal progenitor genes (e.g., nestin), whereas tumors of mesenchymal subtype may be more sensitive to viruses in which replication is driven by the inflammation-activated COX-2 or CXCR4 promoter [62,63,64].

### 3.3. Innate Anti-Viral Responses

Upon cell entry, antiviral host defenses may be activated that counteract a productive lytic cycle and progeny production. There is a plethora of innate sensors that could lead to clearance of the infected cell and halt the viral spreading, leading to resistance to OV therapy. As soon as the cell is infected, the viral pathogen-associated molecular patterns (PAMPs) are sensed by pattern recognition receptors (PRRs). These PRRs include Toll-like receptors (TLRs) [65], RIG-I-like receptors (retinoic acid-inducible gene-I-like receptors, RLRs) [66], C-type lectin receptors (CLRs) [66], oligomerization domain containing receptors {(NOD-like receptors (NLRs)} [66], cyclic GMP-AMP synthase (cGAS) [67] and absent in melanoma 2 (AIM2)-like receptors (ALRs) [68]. The recognition of the viral PAMPs from the host PRRs results in interferon type I (IFNα, IFNβ, IFN-ε, IFN-κ, IFN-ω, IFN-δ, IFN-ζ and IFN-τ) and interferon type III (IFN-λς) production, as well as the expression of interferon stimulated genes (ISGs) and other proinflammatory cytokines and chemokines [69,70].

Although the aforementioned PRRs have been extensively studied, not many studies have attempted to correlate their overexpression in tumor cells with OV resistance; but rather with the aftermath of the recognition, the antiviral interferon pathways. However, a few studies have implicated the cytosolic DNA sensing pathway to oncolytic herpes virus-1 resistance (see below). The main sensor of dsDNA in the cytoplasm is the cGAS, which recognizes dsDNA viruses and reverse transcribing RNA viruses like HIV-1. As soon as cGAS is activated, it synthesizes cGAMP which activates the adaptor protein stimulator of interferon genes (STING) [71]. Stimulation of STING leads to the activation of IRF3 and NFκΒ [71]. Interferon gamma inducible protein 16 (IFI16) is another sensor of dsDNA that signals via STING to activate IRF3 and NFκΒ resulting in IFNβ production [72].

Ultimately, viral detection by the aforementioned sensors will lead to the activation of host defenses such as the production of type I and type III interferons. These have distinct receptors, however, both activate a signaling cascade via receptor-associated protein tyrosine kinases Janus kinase 1 (JAK1) and tyrosine kinase 2 (TYK2), which activate the activator of transcription 1 (STAT1) and STAT2, which subsequently form a complex with the IFN regulatory factor 9 (IRF9), the ISGF3 complex [73]. This complex translocates to the nucleus, resulting in the expression of more than 300 ISGs and pro-inflammatory molecules and establishing an anti-viral state in the infected cell [74,75]. The cytokine and chemokine milieu produced by the infected cell also acts in a paracrine manner to induce an ISG-mediated anti-viral state in the (uninfected) adjacent cells. Some of these ISGs, such as GTPase myxovirus resistance 1 (MxA), ribonuclease L (RNaseL) and protein kinase R (PKR) have direct antiviral activity. For instance, MxA monomers reside in the cytoplasm and upon binding to viral components can degrade them [76]. PKR regulates a plethora of signaling pathways and its role in antiviral response and inhibition of host translation is considered crucial upon virus infection [76].

The anti-viral IFNs are major determinants of OV efficacy. Many OVs exploit the IFN pathway defects to successfully replicate in tumor cells. For instance, it has been shown that STING pathway is correlated with oncolytic herpes virus-1 resistance (see below). However, new evidence shows that this advantage in viral replication may not correlate with tumor eradication in vivo [77]. This may be explained by the inability of oncolytic viruses to induce immunogenic cell death in STING-deficient tumor cells, thus hampering the induction of innate and adaptive immunity [78]. Identifying specific defects in the IFN pathway that may ‘assist’ the viral replication without harming the induction of antitumor immunity, could lead to identification of predictive biomarkers for OV sensitivity.

### 3.4. The Autophagic Response to Viral Infections

There is growing evidence that the role of autophagy in the infectious cycle of many viruses is critical. Autophagy is an evolutionary conserved adaptive process in which the cells attempt to maintain their homeostasis [79,80]. It can be triggered by different types of stress, such as hypoxia, nutrient deprivation and infection [81]. The role of autophagy is to remove detrimental cytosolic material such as protein aggregates and damaged organelles. During this process, a phagophore engulfs cytosolic material to form an autophagosome, which subsequently fuses with a lysosome to degrade its cytoplasmic content [81].

Autophagy is activated by the infected cell to degrade and remove the virions from the cell, a process called xenophagy [82]. TLRs, RLRs and cGAS signaling pathways, among others, lead to autophagy activation to enhance the interferon production and create an anti-viral milieu [83]. Notably, plasmacytoid dendritic cells (pDCs) which lack the autophagy protein 5 (Atg5) showed decreased TLR7-dependent IFNα and IL-12 production after VSV and Sendai virus infection, indicating the importance of autophagy for mounting an anti-viral response [84]. Additionally, autophagy was shown to have anti-viral effects against Sindbis virus and Rift Valley Fever Virus infection [85,86]. On the other hand, many viruses have developed mechanisms to exploit autophagy in favor of their viral replication. For example, herpesvirus and dengue virus were shown to enhance autophagy to promote cell survival in order to establish a successful infection and enhanced viral replication [87,88]. Moreover, rapamycin, an autophagy inducer, was shown to increase the viral replication of various oncolytic viruses in tumor cells, including adenovirus, reovirus, poliovirus, herpes virus, NDV and myxoma virus [89,90,91,92,93,94]. In line with these findings, it was shown that knocking out two key autophagy genes (ATG5 or ATG10) impaired virus-induced lysis of cancer cells by a modified oncolytic adenovirus (delta24-RGD) [95]. Furthermore, co-treatment with Everolimus, a rapamycin derivative, and delta24-RGD enhanced autophagic dependent cell death in an in vivo glioma model [96].

The double-sided role of autophagy in oncolytic virus efficacy has captured the attention of the research community and it has been extensively reviewed [97,98]. The results thus far suggest that, for certain OVs, the tumor cells’ ability to activate autophagy can contribute to the degree of viral replication, and ultimately the therapeutic efficacy of the viral treatment.

## 4. Potential Biomarkers for Sensitivity to Oncolytic Viruses

The different OV strains employed in GBM immunotherapy utilize different cell entry receptors of entry and their cell killing mechanisms are distinct from each other. Furthermore, each OV strain triggers the host responses in diverse ways. Available in vitro and in vivo data provides numerous leads to pathways and molecules involved in OV sensitivity or resistance. OV trials are increasingly incorporating trial-associated (immune) monitoring studies to gain insight into the in situ mechanisms involved in clinical OV therapy. Such valuable data may yield relevant information for identifying potential biomarkers related to response to OV therapy in GBM.

### 4.1. Oncolytic Herpes Simplex Virus

Oncolytic HSV-1 (oHSV) is an enveloped double-stranded DNA virus that belongs to the alpha-herpesvirus subfamily [99]. It is a neurotropic virus and therefore requires engineering for tumor-restricted replication [100]. Modified oHSV-1 variants that have been tested in glioma patients are G207, G47Δ, HSV1716 and rQNestin-34.5 [9]. Safety and feasibility of local oHSV injection in GBM was shown in two phase 1 trials testing G207 and HSV1716 [7,8]. However, in both studies viral replication was detected in only a few patients; 3 out of 6 and 2 out of 12 patients, respectively. These results suggest that the replication of the virus was restricted in some patients. In addition, seroconversion was observed in some patients indicating that the antiviral immune response may have contributed to the rapid clearance of the virus [7,8]. In an effort to understand these restricting mechanisms, Peters et al. studied G207 infection in vitro and found glioma stem cells (GSCs) to be non-permissive to infection [101]. G207 virus contains mutations in both copies of the γ34.5 gene to prevent neurovirulence, however, in GSCs this deletion results in a translational shut down preventing the production of progeny virions [101]. Other oHSV-1 variants designed to express the γ34.5 protein under a tumor-specific promoter such as the rQNestin-34.5, might enhance the oncolytic activity of the virus in GSCs [10].

Another modified HSV-1 is Talimogene laherparepvec (T-VEC) which is the first Food and Drug Administration (FDA) approved oncolytic virus and is indicated for treatment of patients with advanced melanoma [102]. Numerous clinical trials have employed T-VEC, however, no biomarkers for response have been described thus far. In a clinical study in melanoma patients, a favorable outcome was observed in a subgroup of patients with unresectable Stage III or IV M1a disease [103]. Recently, an in vitro study in melanoma cell lines revealed that STING expression can restrict T-VEC-mediated oncolysis and loss of its expression may confer sensitivity to oncolysis [104].

### 4.2. Oncolytic Adenovirus

Adenoviruses are non-enveloped double-stranded DNA viruses [59]. Oncolytic adenoviruses have been extensively explored and utilized in many clinical trials against several cancers [105]. Conditionally replicating adenoviruses (CRAds) have been modified in diverse ways to target oncogenic pathways frequently mutated cancers such as the retinoblastoma (Rb) or the p53 pathway [58,106]. Dl1520 (ONYX-015) was the first CRAd tested in a phase I clinical trial for recurrent gliomas, in which the safety of local peritumoral injection of the virus was shown [107]. Several possible selectivity mechanisms have been proposed for dI1520, including p53/p14ARF defects, aberrant late mRNA transport and cell cycle disruption, all of which may relate to the functions of the early viral E1B-55k gene which is deleted in this virus [11]. Whether any of these factors can serve as biomarkers for dI1520 response has not been evaluated.

The results of another phase I clinical study against recurrent malignant gliomas using the CRAd delta24-RGD (DNX-2401) were recently published [20]. This OV was engineered to selectively replicate in tumor cells with dysfunctional Rb pathway, which is the case in approximately 80% of GBM tumors [12,58]. Impressive anti-tumor effects were found, with 17% of the treated patients surviving beyond 3 years [20]. Another early clinical trial with this CRAd was conducted in our institute in patients with recurrent GBM (NCT01582516). A subgroup of patients revealed high concentrations of different cytokines in post treatment CSF samples, indicating that delta24-RGD can induce an inflammatory microenvironment, which is potentially key for its therapeutic efficacy [108]. Whether specific cytokines can serve as biomarkers for response requires further investigation.

Very few studies have focused on elucidating the resistance mechanisms in the non-responder patients in oncolytic adenoviral trials. In one study, it was demonstrated that the IFN signaling pathway was upregulated in Ad5/3-Δ24-resistant ovarian tumors compared to untreated tumors [109]. Moreover, the authors showed that the MxA, an ISG which is induced by IFN type I or type III signaling could provide a predictive marker for resistance to oncolytic adenoviral therapies [109,110].

Of great interest are the studies that have focused on the pre-treatment immune status of patients receiving oncolytic adenovirus. Specifically, it was shown that chronic inflammation was a negative predictive marker for response to oncolytic adenovirus therapy in different types of cancer [111]. Furthermore, high-mobility group box 1 (HMGB1), a nuclear protein secreted by immune cells and which is associated with a pro-inflammatory state and immunological cell death, could serve as a predictive and prognostic marker for oncolytic virotherapy with adenoviruses. The study suggested that patients with low serum HMGB1 have more robust anti-tumor responses after oncolytic adenovirus therapy [112]. It was hypothesized that a higher pro-inflammatory state as measured by HMGB1, leads to inhibition of viral replication. These results may suggest that the use of immunosuppressants for a limited amount of time during post virus administration, may improve response rates in this subgroup of patients.

Lastly, in a study using pancreatic cell lines it was shown that high expression of cyclin D1 enhances delta24-RGD-induced cytotoxicity [113]. Cyclin D1 activates the cyclin-dependent kinase 4 and 6 (CDK4 and CDK6), which then phosphorylates the Rb protein resulting in cell cycle progression. Over-expression of cyclin D1 has been observed in many cancer types like head and neck squamous cell carcinomas (HNSCC), pancreatic and breast cancer [114]. In GBM, the highest expression of cyclin D1 was observed in the proneural subtype, which may suggest that this subtype would benefit more from delta24-RGD treatment.

### 4.3. Oncolytic Retrovirus

Replication-competent retroviruses are a relative newcomer to the OV field. These are single-stranded RNA viruses. Originally, retroviruses were applied in gene therapy approaches, however, it was later shown that replication competence of retroviruses can provide a powerful tool for gene delivery of anticancer agents in tumors [115]. Vocimagene amiretrorepvec (Toca 511) is a replicating γ-retrovirus derived from murine leukemia virus and is engineered to encode a yeast cytosine deaminase (CD) gene [116]. In the presence of the prodrug 5-fluorocytosin (5-FC), CD converts 5-FC to the potent anti-cancer drug 5-fluorouracil (5-FU). The results from a phase I clinical trial for recurrent high-grade gliomas showed that the median survival of the patients (*n* = 53) was 13.6 months with six patients showing complete response [21]. However, in a subsequent phase III study in 403 patients, clinical endpoints were not met [117].

New results from the earlier phase I clinical trial (NCT01470794) were recently published demonstrating that 86% of the patients that lived >2 years had neoantigens deriving from IDH1, PI3K3CA, EGFR, SYNE1 genes. Interestingly, only 26% of the patients with <2 years survival had neoantigens arising from these genes, suggesting that neoantigens arising from driver genes may support Toca+5-FC therapy response. Moreover, the numbers of M0 macrophages and NK cells at the tumor site at the time of treatment were associated with poor response [118]. It is conceivable that these cells contributed to clearance of the virus before its therapeutic effect could take place. Further investigation is needed to establish if the immune composition could serve as a predictive marker and whether this is also the case for other oncolytic viruses.

### 4.4. Oncolytic Measles Virus

Oncolytic measles virus (oMV) has been applied in many Phase I/II clinical trials against numerous types of cancers including ovarian cancer, pancreatic cancer and glioblastoma (GBM) [119]. MV is a single-stranded, negative-sense RNA virus that belongs to the Paramyxoviridae family [120]. The entry of MV is mediated by the attachment of the viral Hemagglutinin (H) protein to three known cell surface receptors; the complement regulatory protein CD46, the signaling lymphocyte activating molecule (SLAM) or nectin-4 [121]. The wild type strains of MV mainly bind to the SLAM receptor, the attenuated MV Edmonston’s (MV-Edm) vaccine strains enter through CD46 receptor, while nectin-4 can be used by both wild type and Edm strains [121].

The attenuated MV strains have revealed tropism for infecting and killing glioma cells, due to the overexpression of the entry receptor CD46 on the cell surface of these cells [13,122]. Although CD46 is abundantly expressed on glioma cells, facilitating efficient infection, some glioma cell lines show resistance to oncolysis after the viral entry, indicating that other processes can affect its oncolytic efficacy [123].

Indeed, a recent study pinpointed the expression of the interferon-induced transmembrane protein 1 (IFITM1) gene as the responsible ISG for restricting oMV replication in transformed human mesenchymal stromal cells [124]. Additionally, in another study researchers screened eight sarcoma cell lines and found that five of them were susceptible to the oMV. The resistance in the three remaining sarcoma cell lines was attributed to the upregulation of the RIG-I and IFITM1 mRNA expression. Interestingly, it was found that resistance could be broken by increasing the multiplicity of infection (MOI) in combination with the pro-drug 5-FC [125].

The fact that oMVs are sensitive to antiviral responses was also highlighted by a translational study from Kurokawa et al. [123]. Specifically, it was shown that mice bearing GBM tumors with defective interferon pathway were more responsive to oMV treatment, producing 387-fold higher infectious progeny virions compared to mice bearing GBM with intact interferon pathway. Moreover, gene expression analysis of tumor samples from GBM patients treated with oMV (NCT00390299) showed an inverse correlation of ISGs expression and viral replication [123].

Taken together, evidence suggests that IFITM1 expression may be a biomarker for resistance to oMV virotherapy for GBM patients and could help stratify the patients in oMV trials. Further research needs to be performed in other tumor types to investigate if this is a pan-cancer biomarker for oMV response.

### 4.5. Newcastle Disease Virus (NDV)

NDV is an avian paramyxovirus with a negative-sense, single-stranded RNA enclosed in its viral envelope. It was thought that some NDV strains have oncolytic properties by taking advantage of the inability of the tumor cells to elicit an anti-viral response due to deficiencies in IFN pathway [14]. Therefore, the search for markers of resistance to NDV-mediated oncolysis have focused on the antiviral pathways.

Krishnamurthy et al. showed that NDV susceptibility was linked to impairment of the type I interferon pathway [126]. Fibrosarcoma cells that were susceptible to NDV infection were unable to induce IFN-β production [126]. Specifically, the STAT1 and STAT2 phosphorylation was significantly reduced in the permissive tumor cells, resulting in reduced expression of ISG mRNAs and IFN-β [126]. In an approach to overcome resistance, Zamarin et al. engineered an NDV variant expressing an IFNα-antagonist, which demonstrated enhanced oncolytic activity in melanoma cell lines compared to the NDV strain without the IFN-antagonist [127].

In contrast with these findings, Mansour et al. revealed that the human non-small-cell lung cancer cell line A549 was susceptible to NDV oncolysis, despite the production of high levels of type I IFN response. It was proposed that the restriction mechanism in NDV oncolysis was based on the expression level of the anti-apoptotic protein Bcl-xl, where over-expression of Bcl-xl correlates with increased sensitivity to NDV [128]. Moreover, it was recently demonstrated that STAT3 inhibition suppresses immunogenic cell death (ICD) by NDV in melanoma tumor cells [129]. Interestingly, Bcl-xl is one of the target genes of active STAT3 [129].

Another potential marker for NDV cytotoxicity is the status of the autophagy pathway. Meng et al. showed in U251 glioma cells that NDV exploits the autophagic machinery to increase its replication. In this study, inhibition of BECLIN-1 or ATG5 gene, which are critical for the autophagosome formation, led to reduced production of NDV [130]. A more recent study showed autophagy modulators act as sensitizers for NDV in drug-resistant lung cancers [131]. On a different note, Puhlmann et al. showed that the oncogenic protein, Rac 1, is essential for NDV replication and it could confer sensitivity to viral replication [132]. Rac1 is a Rho GTPase protein and is involved in processes like cell proliferation and cytoskeleton organization [133]. In GBM, it has been shown that Rac1 is important for maintaining the stemness of GSCs, which may suggest that NDV could potentially be utilized to target the treatment-resistant cancer stem cell clones within GBM [134].

### 4.6. Mammalian Orthoreovirus (Reovirus)

Wild type reovirus has been tested clinically for various types of cancer including GBM, both by local and systemic administration [135,136]. Reovirus is a non-enveloped virus with 10 segments of dsRNA enclosed in its capsid. The main receptor that wild type reoviruses use to enter the cells is the junctional adhesion molecule A (JAM-A) [15]. Van den Hengel et al. tested a panel of primary GBM cell cultures, showing that they exhibit a large intertumoral variability in JAM-A expression, suggesting that reovirus efficacy may be hampered in low JAM-A expressing tumors [56]. Recently a second receptor was shown to mediate reovirus infection in the central nervous system (CNS), the Nogo receptor NgR1 [137]. The NgR1 expression in GBM cell lines has been established in various studies, however in a recent study it was shown that the NgR maturation, and thus expression to the cell membrane, is inhibited by transforming growth factor-β (TGF-β) 1, which is highly expressed by GBM cells [138,139,140]. The cell surface expression of NgR1 in primary GBM cells is yet to be elucidated.

Reovirus’ natural tropism to replicate and kill tumor cells makes it an ideal candidate for oncolytic virotherapy. Early on, researchers attempted to elucidate the mechanism of tumor selectivity. It was found that the constitutively active Ras signaling pathway potentiates the reovirus replication via inhibition of PKR [141]. Additional studies have supported that Ras pathway activating mutations enhance reovirus replication [142,143]. Contrary to these findings, Twigger et al. showed that reovirus oncolysis does not depend on the status of EGFR/Ras/MAPK pathway in squamous cell carcinoma primary cell lines [144]. Another factor involved in host cell sensitivity to reovirus is determined by the levels of inhibitors of proteases, such as cathepsin B, that are required to disassemble the virus in the cytoplasm. Inhibition of these proteases restricts disassembly and inhibits viral replication [145].

In a clinical phase I trial for high grade gliomas and brain metastasis, the safety of reovirus after intravenous injection prior to brain surgery was demonstrated. The reovirus capsid σ3 protein and the virus RNA detection in tumors varied between the nine trial patients and correlated with the high proliferation index of the tumor cells (Ki67 expression) [136]. The efficacy of reovirus was also evaluated in a clinical trial for non-small cell lung cancer (NCT01708993), in which reovirus (Reolysin) was injected intravenously. A post-hoc analysis of the data obtained from this clinical trial showed a favorable trend for patients with p53 and EGFR mutations [146]. Another clinical trial (NCT01199263) testing oncolytic reovirus in ovarian cancer patients did not show any clinical benefit and authors stated that one explanation could be that only 20% of the ovarian cancer patients harbor Ras mutations [147].

### 4.7. Oncolytic Poliovirus

Poliovirus is an enterovirus in the Picornaviridae family of single-stranded RNA vi-ruses. A non-pathogenic oncolytic variant was engineered by replacing the internal ribo-somal entry site (IRES) of the poliovirus type 1 (Sabin) vaccine strain with the human rhi-novirus type 2 IRES (PVS-RIPO). The affinity of poliovirus for its cellular receptor CD155, which is upregulated on GBM cells, provides a unique opportunity target these tumors [16]. This approach demonstrated potent anti-glioma activity in mouse models and led to translation to clinical investigation for GBM [17,148]. Impressive results were obtained in a clinical phase II trial testing the recombinant nonpathogenic polio–rhinovirus chimera (PVSRIPO), oncolytic virus in 61 patients with recurrent GBM. Twenty-one percent of patients were still alive 36 months after initiation of treatment [149]. The source of this striking response in a subgroup of patients has yet to be elucidated. However, intriguingly, transcriptomic analysis revealed a correlation between low tumor mutational burden, tumor-intrinsic inflammation, and improved survival after PVSRIPO or anti-PD1 immunotherapy in recurrent GBM patients [150]. Further studies are required to confirm whether these characteristics hold predictive biomarker potential.

### 4.8. Oncolytic Parvovirus H-1

Oncolytic parvovirus H-1 is a single-stranded DNA rodent protoparvovirus 1 virus [18]. The lack of pre-existing immunity in humans makes parvovirus an interesting oncolytic virus to explore in the clinic [151]. Oncolytic H-1 parvovirus was administered intratumorally and intravenously in recurrent GBM patients in a phase I/IIa trial [152]. Robust immune response in terms of high infiltration of cytotoxic T cells was observed in the oncolytic parvovirus patients in this first study. Furthermore, in peripheral blood of the patients, specific T cell responses against glioma and viral antigens were detected [152]. Unlike for many other oncolytic viruses, antiviral type I interferon responses are not evoked by parvovirus infection [152]. Interestingly, parvovirus infection of GBM cells is followed by cathepsin B upregulation [152]. Inhibition of cathepsin B protects the glioma cells from parvovirus oncolysis, highlighting cathepsin B’s importance in the parvovirus infection [153].

### 4.9. Oncolytic Vaccinia Virus

Vaccinia virus is a double-stranded DNA virus that belongs to poxvirus family [154]. Its natural tropism to enter the CNS has made it an attractive candidate for systemic delivery in GBM patients [155]. A phase I/II clinical study for GBM patients is currently ongoing (NCT03294486), in which patients are treated systemically with the vaccinia virus TG6002 [156]. Different strains of oncolytic vaccinia virus have been applied for other types of cancer as well and researchers tried to identify predictive response markers [157]. Zloza et al. identified the inhibitory molecule immunoglobulin-like transcript 2 (ILT2) on the cell surface of T cells, as a potential biomarker for vaccinia virus immunotherapy in melanoma patients. Increased expression of ILT2 on T cells was associated with poor response to oncolytic virotherapy using vaccinia virus [157]. Another study attempted to identify biomarkers associated with resistance to vaccinia virus therapy in hematological malignancies [158]. Genes involved in the ubiquitination pathway, DNA damage response and antigen presentation, among others, were identified and associated with resistance to vaccinia virus-induced oncolysis [158].

## 5. Development of Personalized in Vitro Models for OV Selection

It can be concluded that the interplay between the OVs and tumor cells is very complex and that defining a single biomarker or set of biomarkers predicting efficacy of a specific OV may not be achievable. An alternative approach is the development of patient-specific assays to screen a set of OVs and identify the optimal OV for a particular patient. Such a predictive assay would need to provide information on the efficacy of OV infection, replication, and oncolysis as well as on the immune response that is mounted by the OVs.

At present, in vitro culture models from patient-derived tumors have become the gold standard in drug development research for GBM. Our group has developed a preclinical screening system based on patient-derived low-passage cell cultures under serum-free conditions for preserving the molecular genetic make-up of the parental tumors [159,160]. Such screening activities have for example led to the identification of viral sensitizers which enhance the oncolytic activity of delta24-RGD in GBM cells [161]. The screening system was also applied to assess the efficiency of infection, replication and cell killing by four different OVs on a panel of primary glioma cell lines, which revealed tremendous intertumoral heterogeneity in viral sensitivities [56] (and unpublished data). Such panels of molecularly characterized cell cultures may also help identify new markers of sensitivity or resistance to tested OVs.

To gain insight in the relationship between oncolytic efficacy and immune stimulation, a co-culture model of glioma cells and (autologous) immune cell populations, could potentially provide useful insight on the immune response that is triggered after treatment with different OV candidates, as well as on the relationship between infectivity, oncolysis and immune activation. For instance, a co-culture of macrophages and delta24-RGD-infected (and permissive) GSCs revealed a shift of the tumor-supportive macrophages M2 to the pro-inflammatory M1 [108]. Such approaches are also being taken for other forms of cancer. A platform has been established for cancers like colorectal and non-small cell lung in which tumor organoids were co-cultured with autologous T cells derived from the peripheral blood of the patient [162]. With such model systems, the T cell-mediated cell killing could be evaluated for individual patients after infection with different OVs [162].

However, the establishment of such primary GSC or organoid-immune cell co-cultures is time-consuming and may not yield a robust OV therapy recommendation within the required timeframe. Furthermore, with serial passaging of primary glioma cells, the diverse clones that characterize the GBM tumor cannot be maintained [59]. The establishment of ex vivo 3D tumor model systems directly from fresh tumor tissue may therefore offer a more attractive approach for performing OV screens on the heterogeneous landscape of GBM as such models still retain architecture and cellular composition of the original tumor, including the presence of immune cell infiltrates. We previously reported that fresh tissue derived organotypic multicellular spheroids (OMS) offer a versatile system for studying OV infection, replication and tissue penetration [163]. Similarly, fresh GBM tumor slices have been employed to assess oncolytic myxoma virus efficacy [164]. Other approaches being developed include the culture of fresh tumor cells in slices, on matrices and in microfluidic systems [165,166,167]. Culturing fresh tissue also has limitations, since the culturing methods generally favor the tumor cells and not the immune cells [168]. Identification of culture conditions supporting all cell populations would also offer an improvement to these models.

Efforts in the field to generate 3D models from fresh tissue under culture conditions that support and recapitulate the unique immune tumor microenvironment are expected to facilitate investigations into both the dynamics of viral infection and replication in tumor cells as well as the effects thereof on local immune responses [169]. Such systems may in the future offer a tool to screen multiple OVs for a specific patient and select the optimal viral treatment within a clinically-relevant timeframe.

## 6. Conclusions

With the translation of oncolytic virotherapy to clinical trials for GBM patients, impressive responses have been documented in small subgroups of patients. To increase these response rates, better understanding of factors affecting viral replication, oncolysis and subsequent immune activation is required for each of the OVs under development. Studies in clinically-relevant in vitro and in vivo models as well as trial-associated immune and tumor monitoring studies are crucial for defining these factors and are expected to offer leads for stratification of patients in future OV trials.

**Figure 2:**
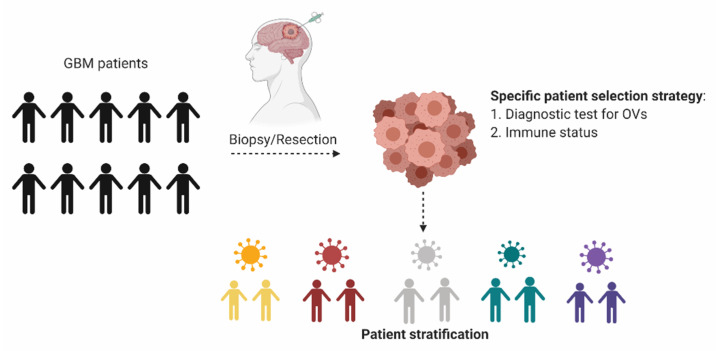
Personalized oncolytic virotherapy. In an envisioned personalized OV trial, resected tumor material from each patient would be used for diagnostic tests to identify the most potent OV, in terms of oncolysis for the particular tumor. Additional tests to determine local and/or systemic immune status after OV infection could further aid in stratifying the patients based on their unique tumor microenvironment and immune status. Created with BioRender.com.

Based on current literature reviews, we have identified factors related to the sensitivity of GBM tumors for specific OVs including the expression of the viral entry molecules, activation state of (cell cycle) signaling or autophagy pathways and the induction of specific antiviral signaling pathways that clear the virus infection (Table 2). These individual markers of sensitivity or resistance may together yield predictive profiles. However, further investigations are needed to shed light on the interplay between oncolytic activity on the tumor cells and the immune system. Ultimately, the complexity of these interactions in a background of a heterogeneous tumor and interpatient immune status variations, may require development of personalized ex vivo models to aid in identifying the most promising OV for a specific patient. The convergence of these developments toward applicable tools will enable classification of each GBM patient as sensitive or resistant to specific OVs. Ideally, future clinical trial design will incorporate more than one OV in parallel arms, such that patients can be stratified to the OV that best matches their tumor properties and/or immune status (Figure 2). Such a selection and stratification approach is expected to significantly improve response rates in OV trials for GBM patients.

## Figures and Tables

**Table 1:** Characteristics of the most commonly used Oncolytic viruses (OVs) in glioblastoma multiforme (GBM) clinical trials.

Family	Genome	OV Examples	Genetic Engineering	Entry Receptor	Tumor Specificity
		HSV1716	ICP34.5-deleted	HVEM, 3-O-sulfated heparin sulfate and nectin-2	Defects in the p16/Rb, PKR or interferon pathways [7]
**Herpesvirus**	dsDNA	G207	ICP34.5 and ICP6 -deleted mutant oHSV	HVEM, 3-O-sulfated heparin sulfate and nectin-2	Defects in the p16/Rb, PKR or interferon pathways [8]
		G47Δ	ICP34.5, ICP6 and α47 -deleted mutant oHSV	HVEM, 3-O-sulfated heparin sulfate and nectin-2	Defects in the p16/Rb, PKR or interferon pathways [9]
		rQnestinHSV-1	ICP34.5-deleted mutant oHSV, in which γ134.5 gene was reinserted under control of nestin promoter	HVEM, 3-O-sulfated heparin sulfate and nectin-2	Expression of nestin [10]
**Adenovirus**	dsDNA	Onyx-015	E1B-55k and E3B -deleted mutant group C adenovirus	CAR	Defects in p53 pathway, defects in cell cycle, late viral RNA export [11]
		delta24-RGD	24-base pair deletion in the E1A gene and insertion of an RGD sequence in the viral knob	CAR, αvβ3 and αvβ5 integrins	Defects in Rb pathway [12]
**Paramyxoviridae**	(−) ssRNA	MV-CEA	Edmonston (MV-Edm) vaccine strain with insertion of the human carcinoembryonic antigen gene	CD46, nectin-4, SLAM	Overexpression of CD46, defects in the interferon pathway [13]
		NDV	Natural tropism	Sialic acids	Defects in the interferon pathway [14]
**Reovirus**	dsRNA	R124	Natural tropism	JAM-A, Nogo Receptor NgR1	Defects in the Ras signaling pathway [15]
**Picornaviridae**	(+) ssRNA	PVSRIPO	Poliovirus type 1 (Sabin) vaccine with replacement of the internal ribosomal entry site (IRES) with the human rhinovirus type 2 IRES	CD155	Overexpression of CD155 [16,17]
**Parvovirus H1**	ssDNA	Parvovirus H-1PV	Natural tropism	Sialic acids	Defects in interferon pathway, defects in cell proliferation pathways [18]

**Table 2:** Potential predictive markers for sensitivity or resistance to OVs.

OV	Potential Predictive Marker	Effect	Tumor Type/ Cell Line
T-VEC	STING	Resistance	Melanoma cell lines [104]
Adenovirus (Ad5/3-Δ24)	MxA	Resistance	Ovarian carcinoma cell line [110]
	High HMGB1	Resistance	Patients with advanced metastatic solid tumors [112]
(Δ24-RGD)	Cyclin D1	Sensitivity	Pancreatic cell line [113]
Vocimagene amiretrorepvec	IDH1, PI3K3CA, EGFR, SYNE1 Neo-epitopes	Sensitivity	Patients with recurrent high-grade gliomas [118]
	NK cells and M0 macrophage tumor infiltration	Resistance	Patients with recurrent high-grade gliomas [118]
MV	IFITM1	Resistance	Transformed mesenchymal stromal cells [124]
	RIG-I	Resistance	Sarcoma cells [125]
	ISG15	Resistance	Primary GBM cells [123]
NDV	Bcl-xl	Sensitivity	A549 cell line [128]
	Rac-1	Sensitivity	HaCaT A5-RT3 [132]
	STAT3	Sensitivity	Melanoma cell lines [129]
Reovirus (R124)	JAM-A	Sensitivity	Primary GBM cells [56]
	Cathepsin B	Sensitivity	Glioma cell line [144]
	P53 and EGFR mutations	Sensitivity	Patients with non-small cell lung cancer [145]
	Ki-67	Sensitivity	Patients with high grade gliomas and metastatic brain tumors [136]
Oncolytic parvovirus H-1	Cathepsin B	Sensitivity	Glioma cell line [18,151]
Vaccinia virus	Expression of ILT2 on T cells	Resistance	Melanoma patients [155]
	LEF1, STAMBPL1, and SLFN11	Sensitivity	myeloid and lymphoid leukemia cell lines [156]
	PVRIG, LPP, CECR1, Arhgef6, IRX3, IGFBP2, and CD1d	Resistance	myeloid and lymphoid leukemia cell lines [156]
